# Working Right Ways: Investigating Health Practitioners' Perspectives of Challenges and Barriers to Providing Good Foot Care With and for First Nations Peoples

**DOI:** 10.1002/jfa2.70179

**Published:** 2026-07-23

**Authors:** James Gerrard, Shirley Godwin (Badimaya Yamatji), Kim Whiteley (Wiradjuri), James Charles (Kaurna), Vivienne Chuter

**Affiliations:** ^1^ Discipline of Podiatry, School of Health Sciences Western Sydney University Sydney New South Wales Australia; ^2^ Wardliparingga Aboriginal Health Equity Unit South Australian Health and Medical Research Institute Adelaide South Australia Australia; ^3^ La Trobe University Rural Health School Bendigo Victoria Australia; ^4^ Independent First Nations Contributor Warramunga–Wiradjuri Sydney New South Wales Australia; ^5^ First Peoples Health Unit Griffith University Gold Coast Queensland Australia; ^6^ University of Newcastle Newcastle New South Wales Australia

**Keywords:** Aboriginal and Torres Strait Islander Peoples, cultural safety, First Nations Peoples (Australia), foot health, practitioner perspective, systemic racism

## Abstract

**Background:**

Ongoing colonisation of the land now known of Australia includes Western hegemony in biomedical knowledge systems dominating healthcare provision. Racism remains structurally entrenched in Australian systems of health and wellbeing. This has serious implications for First Nations foot health outcomes. The purpose of this work is to empower First Nations‐led codesign of ways of working in foot health research to investigate challenges to attending, and barriers to providing, good foot care with and for First Nations Peoples.

**Methods:**

Aboriginal Participatory Action Research ways of working in this study are documented in previous publication; ‘Working right ways in foot health with and for First Nations Peoples: research method guided and governed by First Nations ways of knowing, being, and doing in cross‐sectional qualitative study design’. First Nations‐led study design used decolonised language in semi‐structured interviewing to talk with consenting participants. Data analysis included all transcripts being anonymised and transcribed verbatim by researchers manually. First Nations‐led ways of doing thematic analysis was agreed upon by all authors.

**Results:**

Two talking points discussed First Nations and non‐Indigenous health practitioners' perspectives of providing good foot care with and for First Nations Peoples: (1) What challenges exist in attending individual/organisational services? (2) What are barriers to providing good foot care? In interviews, themes emerged of relationships, disempowerment, education, and clinical governance. Additionally, racism, distance, and funding were perceived as influencing foot care provision. Although not themed, the constructs of time and their importance, were interwoven through results.

**Conclusion:**

Participants described relationships which were not culturally centred, nor longer term, and which therefore limited trust, as being a challenge to attending foot health services. This research identified service barriers to providing good foot care with and for First Nations Peoples as distance and funding. Compounding barriers was lack of health professional cultural capability education impacting clinical services; this being dependant on individuals' behaviours and knowledges, and services' governance. Further work needs to centre the cultures and leadership of First Nations individuals, families, and Communities in working with and for them to keep feet strong.

## 1 Introduction

### 1.1 Nomenclature

This work includes the nomenclature; Aboriginal and Torres Strait Islander Peoples, First Nations Peoples, and Indigenous Peoples. Neither singularly, nor collectively do they adequately represent the immense diversity of language groups and cultural values across this continent's Traditional Custodians and Sovereign Owners [1, 2]. The authors privilege ways of using terminology that are self‐determined, that communicate diversity and sovereignty, and that minimise use of terms that are imposed. Non‐Indigenous people is the language used to represent and be inclusive of Australians who are not First Nations people [3]. The terms decolonise, decolonisation, and decolonising methodology throughout this work describe being inclusive of First Nations worldviews and holistic conceptualisations of health and well‐being [4], whilst challenging and de‐centring dominant colonial views and divesting colonial power [5]. The term method is very much a Western academic term and is frequently described in this publication as ways of working, not just inclusive of, but led by First Nations ways of knowing, being, and doing.

### 1.2 History

The authorship acknowledges that since invasion, Australia's healthcare system has been dismissive of an ongoing and successful First Nations health paradigm in place since time immemorial.

### 1.3 Statistics

When dealing with statistics, pathology, and reported levels of health, authors acknowledge the important considerations and groundings within which to position discussions that respects the strength and pride of First Nations Peoples [6]. The research does not include deficit discourse, does not assign First Nations Peoples as vulnerable populations, and does not present statistics to obscure the root causes of health inequities [6], namely ongoing colonisation and systemic racism [6, 7, 8, 9, 10, 11, 12, 13].

### 1.4 Referencing

Referencing follows the Indigenous Archives Collective Indigenous Referencing Guidance for Indigenous Knowledges in acknowledging knowledge creation to address and dismantle oppressive systems denying people creation of their own culture [14]. This work privileges Nation, Country, or Language group in the reference list, if that information is provided within the source being cited or is clearly provided [14]. This work does not assume a person's affiliation if it is not stated clearly [14].

## 2 Background

Ongoing colonisation of the land now known of Australia includes Western hegemony in biomedical knowledge systems dominating healthcare provision [15]. It is established race has no basis in biology [16, 17], yet the ramifications of scientific racism continue to leave a legacy of racist ideologies [17], and racism remains structurally entrenched in Australian systems of health [13, 18, 19, 20, 21]. Ongoing colonisation and racism harm First Nations Peoples' health and wellbeing [8, 9, 11, 12, 22], and reduce access to health services [8].

Racism reducing First Nations Peoples' access to and engagement with mainstream health services [23, 24], is reflected by lack of cultural safety having the same impact [25]. Specific to foot health, 2019 systematic review research describes a lack of culturally safe services in podiatry [26]. After this 2019 systematic review, the National Scheme's Aboriginal and Torres Strait Islander Health and Cultural Safety Strategy 2020–2025 which governs foot health care in the land now known as Australia was released as a strategy to eliminate racism from healthcare [27]. ‘Cultural safety’ and ‘racism’ are intertwined and inter‐dependent; this Cultural Safety Strategy explicitly identifies the elimination of racism from healthcare as central to the provision of culturally safe care [27]. Tracking progress however, The Australian Institute of Health and Welfare cultural safety in healthcare for First Nations Peoples monitoring framework sights the Australian Reconciliation Barometer in reporting First Nations racial discrimination by doctors, nurses and/or medical staff nearly doubled between 2014 and 2022 [28, 29, 30, 31].

Racism manifests within the mainstream Australian health system through continual inference of First Nations inferiority [16, 32, 33, 34, 35, 36]. Deficit discourse feeds healthcare professional unconscious biases. These biases impede cultural safety [37] and negatively impact care provision [16, 36, 38], with evidence emphasising harms of race‐based shortcuts in clinical practice [39, 40, 41]. In Australia, racism against First Nations Peoples and cultural safety for First Nations Peoples is not related only to skin colour. Around the world, resultant problems with foot health services also run deeper than such superficial association, but do include Black patients having increased odds of 1‐year amputation regardless of anatomical location [42], discrepancies existing in wound care because of skin tone variations [43, 44, 45], racial disparities in the healing of pressure ulcers [46], and erythema, a sign of the diabetes‐related foot complications of infection and Charcot neuroarthropathy, going unnoticed in darker skin [47, 48]. This is alarming in so called Australia where First Nations Peoples are admitted to hospital with diabetes‐related foot disease and undergo lower limb amputations at disproportionately higher rates than non‐Indigenous people [49, 50, 51], with greater risk of all‐cause mortality after major lower limb amputation compared to non‐Indigenous people [52]. Relating to foot health, cultural safety deficit perpetuates violence against First Nations Peoples; racism, discrimination, decentring of culture, and devaluing of First Nations knowledges leading to poorer health outcomes such as foot ulcerations, foot amputations, limb loss, and premature death. Lack of culturally safe service reduces First Nations access to healthcare [28, 53, 54, 55]; consequently, First Nations Peoples live with poorer health outcomes [55, 56, 57, 58, 59], including poorer foot health outcomes [60].

The purpose of this study is to explore challenges to attending, and service barriers to providing, good foot care with and for First Nations Peoples. This study seeks to inform praxis differently; privileging First Nations lived experiences and voices, to achieve different results; good foot health outcomes with and for First Nations Peoples. In Communities where provision of care, including foot care, is culturally safe, First Nations Peoples access health services more frequently and have better health outcomes; including foot health outcomes [26, 55, 61]. Added to this, previous research on Darkinjung Country describes an uptake of ongoing Community engagement with podiatry services when culturally responsive aspects feature [61]. As a concept, culturally responsive care leads to culturally safer experience [62]. This study aims to contribute to development of safer foot health spaces for First Nations Peoples to approach and use, as judged by First Nations Peoples.

## 3 Methodology

This research is part of a study guided by Indigenous research methodology [63].

### 3.1 Methods

Empowered First Nations ways of working are detailed in the previous publication ‘Working right ways in foot health with and for First Nations Peoples: research method guided and governed by First Nations ways of knowing, being, and doing in cross‐sectional qualitative study design’ [63].

This study is Aboriginal Participatory Action Research (PAR) [64]. First Nations leadership for the study includes local First Nations Peoples representative of place, First Nations Advisory Group, and First Nations ethics approval. In decolonising research [5, 65], relationships underpin work which foregrounded how it may be appraised by the Aboriginal and Torres Strait Islander quality appraisal tool [66]. Methods privileging non‐linear and relational ways of working promoted strength‐based approaches to work and facilitated places of 2‐way learning. Spaces within which work was undertaken were anti‐racist [63].

### 3.2 Study Design and Ethics

First Nations‐led study design used decolonised language in semi‐structured interviewing to talk with consenting participants. Ethical approval was granted by The Aboriginal Health and Medical Research Council (1376/18) and the University of Newcastle Human Research Ethics Committee (approval No. H‐2018 0035).

### 3.3 Study Participants and Selection Procedures

First Nations Advisory Group led communication regarding the study which was more likely to be deemed safe to approach and use by First Nations participants [63]. This aimed to reduce the risk of selection bias where First Nations Peoples would not participate because of an understandable mistrust in research.

### 3.4 Participant Recruitment

Purposive sampling seeking variation in cultural identity and place of work was used to recruit health practitioners working with and for First Nations Peoples supporting foot and lower limb health to the study.

### 3.5 Data Collection

Semi‐structured interviews talked with four First Nations and six non‐Indigenous participants about their perceptions of challenges and barriers to providing good foot care with and for First Nations Peoples. All interviews were conducted in English language. First Nations participants were offered a First Nations member of the research team be present during data collection.

### 3.6 Data Analysis

Anonymised transcripts were manually transcribed verbatim. First Nations‐led ways of doing thematic analysis was agreed upon by all authors [63]. This included non‐Indigenous researcher coding being reviewed by a First Nations researcher to control for biases and any data misinterpretation. Cross‐checking process brought dependability in reasoning and comparison to analysis processes. Open codes were recorded in Microsoft Word. No software was used to generate the coding framework which developed from iterative refinement of themes with regular discussion of data. Methods to identify and analyse common themes within talking points in interviews were inductive reasoning and constant comparison.

## 4 Results

### 4.1 Foregrounding Results

This work is part of a larger study which privileges First Nations‐led ways of working [63]. When presenting results, this work highlights complexity of the data and a story of results describing inter‐connected concepts that are relational to multiple talking points [63]. There is relationality in the way First Nations stories are told. Time and contribution of First Nations Peoples with authentic lived experiences are true gifts to this work and are appreciated as such. First Nations data is demonstrably privileged in this work, presented first, and supported by non‐Indigenous data. Supportive non‐Indigenous input is valuable to this study and provides some direction for non‐Indigenous health practitioners, bringing lived experiences and unlearnings working with and for First Nations individuals, families, and Communities. As much as Western research process positions non‐Indigenous researchers as centred and holding power, non‐Indigenous researchers in this work value the teachings from First Nations advisors of responsibility in deep listening and interpreting stories. True to First Nations‐led ways of working in this study, results are a story; told suffixing ‘First Nations’ or ‘non‐Indigenous’ as representative of participants' standpoints and identities [63].

### 4.2 Demographic Data

Table 1 summarises descriptions of 10 participants. Duration of interviews was between 25 and 63 minutes. The number of First Nations health professionals in so called Australia is limited because of systemic racism [67]. To maintain anonymity, reduced participant characteristic data are published. Four participants were First Nations people, nine were podiatrists, and one participant worked in wound care involving the foot and lower limb. At the time of undertaking work, participants worked in regional and remote settings on the lands now known as Western Australia, Northern Territory, Queensland, South Australia, and New South Whales. Participant's places of employment included Aboriginal Community Controlled Health Organisations (ACCHOs), Aboriginal Medical Services (AMS), Hospitals including High Risk Foot Services (HRFS), Community health centres, and clinical education settings.

**TABLE 1:** Participant characteristics.

Are you Aboriginal and/or Torres Strait Islander?	How long have you been a registered health professional in your field?	On what Country do you work?	In what setting do you work?	How many First Nations patients do you provide foot health care to in terms of overall clientele?
Yes	7 years	Gadigal, Darkinyung, Gumbaynggirr Country	AMS and university	‘Hundreds’
Yes	More than 10 years	Larrakia, Warlpiri, Gurindji, and Ngarinyman Country	ACCHO	‘Many Communities’
Yes	20 years	Lands of the Yuggera, Turrbal, Yugarabul, Jagera, Yugambeh, Kombumerri Peoples	ACCHO and other clinical and university settings. Aboriginal missions	‘The majority’
Yes	22 years	Awabakal Country, and across many other First Nations	Hospital and community sector. GP practices and aged care	‘Approximately 20% of clientele’
No	4–5 years	Yawuru Country	Outpatient community settings, AMS, aged care and renal	‘The majority, possibly 90% of clientele’
No	13 years	Arrernte Country	ACCHO	‘Predominantly Aboriginal and Torres Strait Islander people’
No	33 years	Kaurna Country and Anangu Pitjantjatjara Yankunytjatjara lands	Almost exclusively ACCHO	‘80%’
No	10–11 years	Darkinjung and Wiradjuri Country	ACCHO, land council and university	‘All patients are First Nations Peoples’
No	3–4 years	Larrakia Country	High risk foot service, public sector hospital, inpatient and outpatient care	‘60%’
No	5–6 years	Yawuru Country	Public sector with ACCHOs and RFDS	‘80%–90%’

### 4.3 Scope of Practice

Participants worked in foot health settings with general practitioners, vascular and orthopaedic surgeons, and with a variety of health professions including Aboriginal and Torres Strait Islander Health Workers and Practitioners. Working alongside renal services and Social and Emotional Well Being (SEWB) services, participants supported people living with cardiovascular disease, and chronic diseases including diabetes and kidney disease, and delivered foot health education with and for patients and healthcare staff. Collectively engaging a broad scope of foot health practice, participants were involved in offloading, resource development, wound care, treatment of physical injury, Telehealth, service accreditation, and shared decision making in foot health settings. Participants had lived experiences in referring patients within and between different models of service delivery, and firsthand accounts of challenges and barriers to providing good foot care with and for First Nations Peoples.

Key themes that emerged from talking points are described in Table 2.

**TABLE 2:** Themes.

Talking points	Themes within talking points
Challenges clinicians see for First Nations Peoples in attending individual/organisational services	Relationships
	Clinical safety
	Transport/travel
	Appointments
	Cross‐cultural interface
	Disempowerment
Individual/organisational service barriers for clinicians to providing good foot care with and for First Nations Peoples	Education
	Practitioner cultural capability, cultural humility, power, and bias
	Clinical governance
	Distance
	Funding

## 5 Themes

Talking Point 1: Challenges clinicians see for First Nations Peoples in attending individual/organisational services.

Key quote: ‘I think it does still get back unfortunately to some of that ego, and (health professionals) needing to be a little bit more humble… still being proud of what they've done and their achievements, but that's what maybe their family is there for, to pat them on the back, they don't, they shouldn't necessarily need to get pats on the back from Community or their patients to be honest’

(*First Nations*)

### 5.1 Theme Relationships

First Nations participants identified First Nations health workforce having established relationships with people attending foot care translating to increased service engagement; ‘I've seen a lot when I (a known First Nations practitioner) would go to places, we would see the actual attendance for podiatry foot care exponentially increased’ (*First Nations*). Conversely, it emerged from data analysis that disruptions to service provider/service user relationships presented challenges to attending foot care services; ‘when there was initial change of podiatrist there was… a hesitancy (to attend), because Aboriginal people culturally and Aboriginal Communities place a lot of value on relationships and trust, and that takes time to grow’ (*First Nations*). Results included individual and systems level relationship considerations; ‘it takes time to show the Community who you are, what you are about, and build that trusting relationship. I think that's where we struggle sometimes, in health we want a quick fix, if we're going to spend money or invest in something we want to see the outcomes straightaway, and unless we really focus on building a medium term to long‐term approach that's going to respond to really complex intergenerational challenges, I think we will struggle to build meaningful relationships that facilitate change’ (*First Nations*).

### 5.2 Theme Clinical Safety

The theme of clinical safety presenting a challenge to attending service provision emerged in First Nations data. ‘(In urban, regional, and remote locations and mostly mainstream settings) I come across clinicians (who) don't know the basics… How do you assess a wound? How do you treat a wound? What's the best thing to treat it with? Very rarely do I find anybody besides a podiatrist that can do a Doppler, ABPI, or toe pressures, and also sharp debride’ (*First Nations*). Compounding this challenge is that ‘there wasn't a lot of training that I thought aligned well with working in a remote context in Aboriginal and Torres Strait Islander Communities’ (*First Nations*). ‘Training very much assumed continued podiatric services, maybe continued access to all sorts of other services to help support podiatry or foot care, and I thought it was a significant finding, that we couldn't find too many training or current training (resources) that supported foot care provision, not just for podiatrists, but primary health care providers in general, in a remote context’ (*First Nations*); ‘make the (clinical) training something that the staff could do quickly and easily, but would also be meaningful and have a benefit for the client’ (*First Nations*).

### 5.3 Theme Transport and Travel

First Nations voices established the theme with ‘things like transport’ (*First Nations*) present as challenges for people attending service provision. This was supported by non‐Indigenous data. ‘I think the transport in coming to locations is often challenging’ (*non‐Indigenous*). ‘I think there is often not enough drivers’ (*non‐Indigenous*). ‘I definitely rely a lot on the Aboriginal Liaison Officers or the Aboriginal Health Workers in the clinics to provide transport into the appointments’ (*non‐Indigenous*). ‘Transport is always, always a big one (challenge) and different First Nations Communities have different levels of vehicle ownership’ (*non‐Indigenous*), and ‘it's not like a city environment in terms of frequency of (public) transport. So (people) often have to organise appointment times around those services which is a challenge’ (*non‐Indigenous*). So ‘for those who are coming down to town, so people who are coming from that sort of rural or remote area down to the city, there's huge (challenges) still’ (*non‐Indigenous*). ‘Some areas, some Communities, I'll only travel to once every 3 months. And, you know, if people are travelling on those days, then they won't be able to see me on that date and then it'll be 6 months before I get to see people again’ (*non‐Indigenous*). ‘Even just notifying people that I'm coming… with the lack of phone reception and people's numbers changing fairly frequently and things like that too’ (*non‐Indigenous*), that creates challenges to attend service provision.

### 5.4 Theme Appointments

First Nations voices raised lack of drop‐in availability as a challenge in attending service provision. ‘More in government control where they don't have a drop in policy’ (*First Nations*), ‘what you find is if people can't come because of cultural obligations, obviously, (many services) don't understand the strength or the super importance of cultural obligation, (i.e.,) funeral attending, that's more important than going to get your feet done’ (*First Nations*). ‘Often the government (services) wouldn't have a drop in policy, they wouldn't allow that to happen, and then you would just get people not turning up and then there would be massive gaps in the system’ (*First Nations*). Drop‐in availability ‘can also allow… for (people) to meet you and have that experience’ (*First Nations*), and ‘I think the good thing about podiatry, it probably lends itself I think to be more drop in, or more spontaneous, in the sense that you can probably do something pretty quickly. You might not be able to deal with everything, but you can probably still do something fairly quickly to sort of get that job done, so to speak’ (*First Nations*). Non‐Indigenous data supported this theme. ‘Just that structured timing of the way we schedule appointments and that people need to adhere to specific times is often really, really challenging’ (*non‐Indigenous*) and ‘that appointments need to be structured and timed, and not able to be flexible to when people have the opportunity to receive care is challenging’ (*non‐Indigenous*). Challenges to attending were noted in appointment inflexibility favouring service providers, but also in contributing to negative stereotypes. ‘Where (patients) had to cancel or reschedule an appointment, when they've come in the next time they've brought it up and said, ‘Oh, I had to take my Auntie or Uncle to something, or my cousin to this thing’, or ‘I had to go pick up someone’, so that idea of looking after your family and things like that… it's just the way it is. So that's probably one that's… easy to write up as… (people) don't care about their foot health or they don't care about their health because they're not coming to the appointment. But that I don't think is the case. It's about (people's) responsibility to family’ (*non‐Indigenous*).

### 5.5 Theme Cross‐Cultural Interface

First Nations data voiced challenges to attending foot care service provision at its cross‐cultural interface. Ways of working ‘changes from Communities, we've got Communities that are just like an hour down the road from each other but they're so different, and how you would engage with both is different’ (*First Nations*). In addition to recognising diversity, results included service improvements to accommodate cultural constructs, ‘a lot of people living with a wound don't want to talk about it because there's embarrassment, and particularly for our mob it's shame’ (*First Nations*), and results included development of culturally responsive care, particularly remaining culturally humble. ‘Again, it's about that humility’ (*First Nations*). ‘You could tell that (health practitioners) just thought they were so important… bring your levels of your ego down’ (*First Nations*). ‘It can be a bit of an issue where people (health practitioners) are often also not wanting to, they're also the same people that don't want to because they're so structured… that's in the Western model that they've come (from) and they've been so successful (in)’ (*First Nations*).

### 5.6 Theme Disempowerment

Non‐Indigenous data raised challenges in accessing services relating to disempowerment and ‘that sense of ownership of the clinics as well. So, people are much more willing to come in and to actually participate in the service’ (*non‐Indigenous*) where there is Community ownership or First Nations controlled health services. Without it ‘I think that location in which people receive their health care being a clinical location and a very Western structure environmentally, may not always feel safe, or feel comfortable, for people’ (*non‐Indigenous*). There can be ‘insecurity with negotiating that hospital clinical setting, with very tight time frames, and crowded areas where people are coming in and going out very quickly, and there just isn't the time for explanation or to sit down and to talk through things’ (*non‐Indigenous*). ‘Perhaps more than anything else, (there's the challenge)… in the sense of people's own needs and priorities not necessarily being recognised and acknowledged within the hospital system’ (*non‐Indigenous*) and ‘what the plan is going to be? (People) want to know how long they've got to stay there—when they can go home. You know, (people) don't like that open ended ‘you're here until things heal’… and that is quite distressing for a lot of people where there's just so many unknowns, and in an unfamiliar environment’ (*non‐Indigenous*).

Talking Point 2: Individual/organisational service barriers for clinicians to providing good foot care with and for First Nations Peoples.

Key quotes: ‘If I'm honest—sovereignty’

(*First Nations*)

‘I think in some health institutions, there is stereotyping, racism, dismissing of Aboriginal Torres Strait Islander Peoples’ health concerns and worries, and when people aren't fitting into the existing structured fixed health systems that we have, that we provide limited accountability on those health services or flexibility to meet the needs of Aboriginal people’

(*non‐Indigenous*)

‘I don't think there's many health services that are structured… where (First Nations Peoples) can observe what's going on and try and build that trust… so the last couple of hundred years means it's not been a safe space…’

(*non‐Indigenous*)

### 5.7 Theme Education

Within First Nations data, results emerged of lack of formative cultural capability education for health professionals being a barrier to providing good foot care with and for First Nations Peoples. ‘It would be good if you could have even the beginnings of the learnings in university of how you might treat Aboriginal and Torres Strait Islanders, especially in a remote context, and how it would be different’ (*First Nations*). ‘Starting with educating… just the beginnings of something at university to help maybe generate interest (in working in the space), but also support students when they go into practice (having established skills and knowledge)’ (*First Nations*). Non‐Indigenous voice supported this theme. ‘If you don't have that understanding (of First Nations ways) in the first place, that becomes a major, major barrier’ (*non‐Indigenous*). ‘A lot of the barriers that I see are particularly around just lack of understanding’ (*non‐Indigenous*).

### 5.8 Theme Practitioner Cultural Capability, Cultural Humility, Power, and Bias

Listening to First Nations voices, the theme emerged of a barrier primarily linked to cultural capabilities and biases of staff, and then to the resulting level of cultural safety of foot care services being safe to approach and use, as judged by First Nations Peoples. ‘Even if you're a dark skin Aboriginal person going to another Community, that's not your Community, you don't just walk in and it's all just in…’ (*First Nations*). ‘The next time you go, there's that little bit more, where you get to talk with (Community) and there's that snowballing or that getting to know people…’ (*First Nations*) ‘That's a starting point, but a lot of people want to skip that. It's like, no, no, (non‐Indigenous practitioners) just want to get in there…’ (*First Nations*). ‘You've got to do this stuff (develop cultural capabilities) and it's a mistake to try to push that any other way in my opinion. And I think it is a barrier in the sense that that's a requirement, and people don't often understand or feel that; I think that's just one of the issues, and then they wonder… why people aren't coming to see me or… they're not turning up… and I think that's part of that (service barriers to providing good foot care)’ (*First Nations*). First Nations results highlighted ‘there are still some issues sometimes with, in the sense of culture, men's and women's business. I think people (service providers) don't always understand, particularly say some of the podiatrists’ (*First Nations*). Compounding this was inconsistency in staffing of clinical service creating a barrier to providing good footcare where 2‐way learning in cross cultural relationships is lost with staffing turnover; ‘because what often happens is a sort of a turnover of people, lots of fair and good reasons, but then they (staff) start again, you know what I mean? And then they (staff) don't know that person (service user) and they don't probably take the time to, to get to know people’ (*First Nations*). ‘It (taking time to develop cultural responsiveness as a health practitioner) just needs to happen for it (foot care service) to be successful (as judged by the service user), or at least having someone there that's consistent, doing that’ (*First Nations*). ‘We've got all sorts of workforce shortages everywhere’ (*First Nations*) without ‘continued podiatric care available to remote Aboriginal and Torres Strait Communities’ (*First Nations*). Within this theme First Nations data highlighted the need for foot care services to deliver consistent culturally capable presence. ‘So, you've got that someone who can continue care, see people more regularly, probably provide education and support to the staff; so they can provide support and care for foot care as well… provide more sustained education and support to clients in Communities with (living with) diabetes, foot problems, or at risk of diabetes foot problems’ (*First Nations*). First Nations data further described ‘this point of contact then for referrals… someone that the hospital and the client can liaise with when they come back into Community… and you've got sustained care available to mob’ (*First Nations*). Informed non‐Indigenous voices supported and contributed to this theme. ‘I recently have reflected a lot, I'm a young white girl going into Community, often viewed as the expert (by non‐Indigenous people) and… I think that is definitely a barrier… working more with Aboriginal Health Workers and reflecting on my practice, it would help to overcome that’ (*non‐Indigenous*). ‘There is stereotyping, racism, dismissing of Aboriginal Torres Strait Islander People's health concerns and worries…’ (*non‐Indigenous*) and ‘there isn't that appreciation of the practicalities and the priorities (of people)’ (*non‐Indigenous*), compounded by health professionals making ‘assumptions without having that background, and that's an enormous issue’ (*non‐Indigenous*). ‘Another major barrier is that quite often there hasn't been the time taken to bring everybody along on the journey as far as management of foot problems goes’ (*non‐Indigenous*), data identified that often health professionals ‘haven't thought to actually include other members of the family who are going to be involved with that person’ (*non‐Indigenous*), nor thought to ‘explain the sort of the circumstances and the context for it, so then there isn't necessarily the support and the backup’ (*non‐Indigenous*). ‘A lot of different cultural barriers’ (*non‐Indigenous*) emerged in data analysis with participants ‘working across a number of different Nations with a number of different cultures and languages’ (*non‐Indigenous*). Further barriers themed with practitioner cultural capability and bias included that ‘there's often a lack of understanding (on behalf of the service) where English is second or third language or fourth language’ (*non‐Indigenous*), and ‘using inappropriate terminology or talking too fast’ (*non‐Indigenous*). ‘It's coming back down to having that level of communication and understanding’ (*non‐Indigenous*) and working ‘to get a good knowledge base of all those different cultures (where permitted) that (health professionals are) working with’ (*non‐Indigenous*). This included working to learn ‘where people are going to sit in terms of providing, supporting other members of their wider family’ (*non‐Indigenous*) and the ‘principle of Ngapartji Ngapartji (Pitjantjatjara Language) which is, sharing, sharing things’ (*non‐Indigenous*). With respect to service not understanding of people not always having their designated footwear for example, ‘there's this assumption that people don't care because that's happened, when it's quite the opposite, and that then becomes a barrier to provision of further footwear’ (*non‐Indigenous*). Further supportive non‐Indigenous data relating to the theme of practitioner cultural capability and bias were ‘staffing barriers… transient population of staff’ (*non‐Indigenous*) which ‘makes it harder… to communicate effectively with people and to… develop long term plans’ (*non‐Indigenous*).

### 5.9 Theme Clinical Governance

Data analysis revealed real lived experience and understanding that mainstream foot care services are not, and have never been, structured to be safe spaces for First Nations Peoples. First Nations results posited ‘is that care being delivered in a model which is conducive to uptake by the people who need it? And that's not just Aboriginal people, people of other cultural minorities in Australia, people who may not have English as a first language, there are a number of really, really common barriers across the community that you know would benefit from more innovative consideration of how we can provide care in a way that's accessible’ (*First Nations*). ‘(First Nations People) don't want to go to a (mainstream) clinic, they don't want to go to the doctors, because that has a different meaning in… I've had Aboriginal mob tell me plenty of times “I go there because I'm gonna die” rather than “go there to get my foot checked”’ (*First Nations*). This theme continued to emerge with supportive non‐Indigenous data. ‘The Western health care model as a whole can be a bit of a barrier to people of an Indigenous background’ (*non‐Indigenous*) and ‘(First Nations People) don't particularly like going to (hospital) because it's not a particularly friendly place’ (*non‐Indigenous*). Clinical governance barriers included a ‘lack of situational awareness on the part of the service provider’ (*non‐Indigenous*) in ‘providing or finding a location that that Community deem as suitable and safe and appropriate for the foot health care to be delivered’ (*non‐Indigenous*). This was reflected by; ‘the structure of the clinic in terms of what works for that Community, whether it's that Community consultation approach’ (*non‐Indigenous*), ‘and if that can't happen, I think that's a potential barrier to being able to provide effective foot care.’ (*non‐Indigenous*). Data analysis in this theme noted clinical governance ‘limited flexibility to meet needs of First Nations Peoples’ (*non‐Indigenous*) such as ‘lack of knowledge about support systems that are available to help people to get into appointments’ (*non‐Indigenous*). ‘(Services) are too quick to dismiss that people just are too lazy to turn up for appointments’ (*non‐Indigenous*), ‘when they don't turn up, then they'll just discharge them off the rolls without actually going back to work out how that should actually all be organised’ (*non‐Indigenous*). Further barriers themed within clinical governance included no ‘way in place to take the time to get the strategies in place’ (*non‐Indigenous*), ‘different systems with the different organizations… don't always communicate very well’ (*non‐Indigenous*), ‘and limited accountability on those services’ (*non‐Indigenous*).

### 5.10 Theme Distance

First Nations results identified distance as a service barrier for practitioners to providing good foot care with and for First Nations Peoples; ‘remoteness is an issue. So, I think it is a barrier. I think it's about that consistency, people want to know people’ (*First Nations*), so ‘barriers are getting into Community to talk’ (*First Nations*). When ‘very remote, obviously having to drive a long way’ (*First Nations*), ‘a number of Communities across a big distance… I would have thought ideally, you'd probably have more than one podiatrist operating across the region on a full‐time basis to help support each other; help build that rapport in those Communities. Because I think if you can have someone in those Communities longer to help build and then sustain that trust and those relationships with Community, there is more of a benefit for everyone, especially the clients’ (*First Nations*). Supportive non‐Indigenous data contributed to the theme, ‘the main thing, there is just simple distance’ (*non‐Indigenous*). ‘Travel can be a barrier’ (*non‐Indigenous*), ‘just simply getting out there to initially educate and provide those services’ (*non‐Indigenous*) can be a service barrier. ‘Just how much distance there is, and the ability for these resources to get out there, or the government backing these sort of services to be in these remote areas’ (*non‐Indigenous*), was identified and was compounded by the barrier of ‘roads get cut off with weather and things’ (*non‐Indigenous*).

### 5.11 Theme Funding

First Nations results highlighted the theme; ‘the reality is funding’ (*First Nations*). There can ‘not be a lot of services there on Community. So, I think that's a barrier. I think facilities, the funding for those facilities’ (*First Nations*). ‘I think people being able to spend money, how they want to spend it, with what the Community want, rather than people (funding sources) pulling the purse strings or saying, “well, we can give you money, but you have to do this, and you have to do that”’ (*First Nations*). First Nations results included that ‘it can be expensive when we look about the best levels of high‐risk foot clinic, they can involve multiple specialists, even in the surgical field, vascular surgery is a big one, but you can also have… orthopaedic surgery as a part of that’ (*First Nations*) as well as ‘highly specialised podiatrists’ (*First Nations*). In terms of podiatrists, compounding this is ‘a very small pool of people who have had all those years to hone those skills and develop that expertise, so there's a bit of a you know capacity gap there in the podiatry community’ (*First Nations*) and ‘is there capacity or adequate funding to grow that workforce and build that capacity?’ (*First Nations*) So, two big barriers are; ‘investment required to grow the capacity so that there's adequate workforce, (and) adequate supply to meet what we see increasingly as growing demand’ (*First Nations*). Supporting First Nations voices in this emerging theme, non‐Indigenous data included the service barrier of ‘funding and having those services even available in the first place’ (*non‐Indigenous*). ‘There are solutions out there but quite often people don't take the time and the effort to source them’ (*non‐Indigenous*). To this end, ‘support from higher up… to be able to disperse our specialist services to the more remote areas… and to then just be able to build something’ (*non‐Indigenous*), there are instances of ‘getting people care flighted from (Communities) costing the government, $10,000 plus, just for one care flight over and back, for something that potentially could have been sorted if it was caught earlier in that area’ (*non‐Indigenous*).

## 6 Discussion

First Nations and non‐Indigenous participants identified numerous themes when discussing challenges to attending, and service barriers to providing, good foot care with and for First Nations Peoples. Importantly, these themes are relational to each other, as well as to people, and to places. They are not mutually exclusive. Notably, thematic analysis in this study did not identify time; yet concepts of time are entwined throughout themes resulting from both talking points. Whether continual progression of existence, indefinite or a measurable period, an occurrence or sequential relation to past, present, and/or future, time presents as being intrinsic to being able to overcome this study's identified challenges to attend services and service barriers to providing good foot care. Within results, time is identified as required for sitting down to talk, for relationship progression, to get strategies in place, and to source solutions. Time is mentioned in this study by both First Nations and non‐Indigenous participants. Time is described as both needed to be taken and required to be provided. Full‐time basis of work, timeframes, and specific times are mentioned, and structured and timed appointment times discussed. In this study, ‘the next time you go’ to visit a place or person is linked with; future, progression, development, improvement, positivity, invitation, 2‐way learning, and with ways of doing being ongoing. This discussion emphasises that time allows for bringing everybody along on a journey improving one's foot health and doesn't just consider linear time, but also its non‐linear conceptualisation [68].

Challenges in attending foot care services with and for First Nations Peoples identified in results of this work included the status of relationships with service providers, clinical safety, appointment structures, as well as progression at the cross‐cultural interface. This study, as well as other work including comprehensive review privileging First Nations ways of working and authorship [7, 69], establish that different approaches to allocating time may underpin negating these challenges [7, 69]. Integral to providing good foot care with and for First Nations Peoples was relationship building. This requires time for deeper listening [70], for hearing First Nations Voices, for respecting silences [71], and therefore, as identified as needed in this study, for building trust [7, 69, 72, 73]. Relationships are named as a cornerstone of First Nations ways of knowing, featuring centrally in the Aboriginal and Torres Strait Islander Health Curriculum Framework [74] which amplifies First Nations ways of learning delivery of good foot care. Relationships are also privileged in ways of doing, positioned as a shared value underpinning Indigenous Allied Health Australia's (IAHA) Cultural Responsiveness in Action Framework [62], which demonstrates First Nations ways of providing good foot care with and for First Nations Peoples. Taking time also supports 2‐way learning at the cross‐cultural interface. Identified as required in the results of this study, this facilitates practitioners' learning, and in working with and for First Nations Peoples, developing foot care plans centring aspects of service users' culture [75]. This can increase positive health experiences in foot care and make for a progressive, approachable cross‐cultural interface. Practitioner learning empowers service users; listening to and learning from service users may challenge clinical structures, making foot health systems more culturally responsive and making them fit the service user, rather than the service user having to fit the foot health system. Relating to results of this study, taking time to consider foot health systems' roles in service users not being able to attend fixed appointments, and using time in alternative ways to rigid traditional Westernised health care service delivery via 1:1 consultation, may redistribute power [76], reduce gratuitous concurrence [77, 78], and better include family and friends in shared supportive decision‐making regarding foot health [79]. Taking time to slow down thought processes, data interpretation, and clinical decision‐making may reduce race‐based snap judgements and implicit bias negatively impacting health care delivery and outcomes [80, 81, 82].

Themes emerging from both challenges in attending, and service barriers to providing good foot care with and for First Nations Peoples talking points advocate for more formal cultural capability education, more ongoing professional development in cultural responsiveness, and more culturally responsive resources made with and for First Nations Peoples. Within them, themes included strong connections between cultural safety and increased access and attendance. Whilst this is certainly important, this work highlights that cultural safety is not only about peoples' entrance into clinical spaces. It is also about dignity, trust, and reducing harm, even where patterns of attendance do not change. The National Scheme's Aboriginal and Torres Strait Islander Health and Cultural Safety Strategy 2020–2025 [27] is ‘a strategy that aims to eliminate racism from the health system’ [27]. This strategy directs ways of being, knowing, and doing good foot care delivery with and for First Nations Peoples, as judged by First Nations Peoples. It is from this strategy that cultural safety is enshrined in National Law, under which, providing culturally safe racism free care is a legal requirement [83]. In line with this First Nations developed governance, Podiatry Registration Board Professional Capabilities have changed [84]; to govern instigating and maintaining ongoing practitioner cultural capability development. There is clear requirement for health practitioners to upskill themselves. Facilitating this, peak First Nations agency IAHA additionally operate Cultural Responsiveness Training [85], offering learning opportunities for service delivery to improve cultural capabilities. However, despite First Nations‐led cultural safety governance [27], despite First Nations‐led Cultural Responsiveness frameworks and training [62, 85], and despite recent changes to professional capabilities [84], the results of this study show health practitioners still recognise lack of cultural capabilities in the health workforce as a key challenge to attending, and a service barrier to providing, good foot care with and for First Nations Peoples. Systematic review research describes both a lack of culturally safe services in podiatry [26], and a lack of access to culturally safe health care delivered by culturally capable health professionals being perceived as contributing to worse foot and lower limb health outcomes [86]. Research also describes Indigenous Peoples frequently subjected to discrimination within Western healthcare [87], Indigenous Peoples having difficulties accessing health care they judge as culturally safe [87], and health practitioners confident in working at the cross‐cultural interface barely acknowledging key principles of cultural safety; racism, power imbalance, bias, and self‐reflexivity, in perspectives of their individual and organisational cultural capabilities [88]. There are fundamental issues around workforce capacity in relation to culturally capable health practitioners which the results of this study highlight, with the key challenge being how the existing workforce can be trained to align with the new professional capabilities and National Law legal requirements [83]. It remains that individuals need to know themselves, to self‐reflect, and to consider where they stand and where they need to make improvements to better cultural responsiveness and advocacy. At a systems level, advocacy includes profession member organisations such as the Australian Podiatry Association of Australia being lobbied to prioritise, fund, and provide authentic, profession specific, and ongoing First Nations‐led cultural capability continuing professional development. At an individual level, advocacy for example, includes lobbying foot care services to provide adequate patient transport; as raised in this study, ‘lack of’ is rooted in systems of discrimination delivering inequities in social determinants of health and inequality in health outcomes.

Service barriers to providing good foot care with and for First Nations Peoples identified in results of this work included education underpinning practitioner cultural capability and bias, clinical governance, funding, and distance. Results within this work call for non‐Indigenous education in cultural responsiveness to include cultural humility in working with and for First Nations individuals, families, and Communities. That you can never learn everything about another culture, and that there are elements of peoples' culture you may not be allowed to know is imperative [89, 90, 91]. Finite competencies of aspects of diverse cultures should never be an expected end‐goal of the learning. Cultural humility ‘does not have an endpoint or goal; there is no objective of mastering another culture’ [92]. ACCHOs and the Communities which control them provide lived learning experiences to providing foot care, but it is not a non‐Indigenous birth‐right nor professional entitlement to walk into Community and request education to improve. Invitation must be earned. Over time. Then the place‐based learning can begin. Working in First Nations foot care is not an exercise in cultural tourism, a chance to change climates for 3 months, nor an opportunity to become a ‘discoverer’ [93]. If the podiatry profession and health practitioners involved in foot and lower limb health are to reduce barriers to providing foot care with and for First Nations Peoples, then authentic, humble, genuine, effort‐based, continuous learning needs to take place. Whilst discussing cultural safety in this instance as a ‘practitioner issue’, and placing strong emphasis on training, humility, and practitioner capability, this study acknowledges too, that as much as those elements matter, cultural safety is not created through training alone. It is additionally shaped by how services are structured, who holds decision‐making power, and whether Communities have real authority in how care is delivered. Without that, even well‐trained and well‐intentioned foot health practitioners are still working inside systems that can feel unsafe for First Nations Peoples to approach and use.

Results of this study also included First Nations and non‐Indigenous perspectives of funding's coercive control and influence as a service barrier to providing good foot care with and for First Nations Peoples. This recognition gives opportunity to acknowledge that funding shapes what services exist, how they operate, and whose priorities are reflected. In this way, funding sits within the broader question of who holds influence over care. Social determinants of health, funding and resources allocation, and power in decision making, are all underpinned by history and ongoing systemic racism and colonisation. Funding allocations speak to priorities, and in the case of First Nations foot health, describe systemic racism. First Nations Peoples live with disproportionate risks to foot health and disproportionate complications to foot health [60], yet First Nations and non‐Indigenous perspectives reported in this study consider funding for services delivered at Community‐determined locations, by culturally capable health professionals undertaking life‐long learning, or by First Nations staff, to be disproportionately low. In the foot health space this reflects non‐Indigenous power deciding what is important and how much foot health is worth for First Nations Peoples. Systemic racism is clearly identified by funding allocation; ‘There's a difference between “we have no budget for this for this,” and “we didn't allocate any budget for this”’ [94]. Both funding and distance are identified in this work as confounding attending foot care services and foot care service delivery. While results of this work describe distance being a barrier in the context of regional and more remote settings, authorship highlight most First Nations Peoples live in regional and urban settings [95] and that regional and urban ACCHOs recognise transport barriers in mitigating all distances to accessing foot care. In terms of consideration of funding and distance in finding solutions that support foot care on Country [75, 96, 97, 98, 99], examples of how the impacts of distance are overcome with TeleHealth foot care include such service reducing patients' travel burden [97]. Telehealth has more recently been used to facilitate First Nations Peoples staying on Country [97], highlighting that dedicated funding, time, and effort need to be put into finding, implementing, developing, and staffing solutions that support successful First Nations foot health paradigms. Paradigms where people are surrounded by protective factors of wellbeing including, family, kinship, Community, culture, Country, spirituality and Ancestors, and culturally responsive and effective health services and professionals [100].

This study's findings and discussion are informed by First Nations lived experiences and First Nations voices. Findings and discussion described engagement with foot health services improves where First Nations practitioners are involved or where services are Community‐controlled. This was reflected in familiarity and relationships, but it also reflects something deeper: When Community has a role in shaping care, people engage differently. That shift is about ownership and authority, not just connection. Additionally, this study associated foot health workforce with a shortage, and whilst rightly identified, work highlights that continuity of care with and for First Nations Peoples is not only about staffing levels, but that it also comes from cultural presence, trusted relationships, and local knowledges. Based on the results of this study along with other studies of foot health, this work highlights that ongoing change needs to happen to improve foot health services with and for First Nations Peoples. Foot care services, that as described in this study, need to; be funded according to First Nations identified needs, be staffed by culturally responsive people, be placed in locations determined by Communities, be structured by First Nations‐led co‐design, buck the tyranny of distance, include First Nations health workforce, and centre First Nations cultures. Putting non‐Indigenous learning, unlearning, and re‐learning into action negates complacency and complicity, it changes structures and systems. It can shape clinical governance, aligning it with National Law legal requirement [83]. ‘Racism is the biggest public health issue that Australia faces today, and no‐one wants to talk about it or do anything about it’ [101]. The National Scheme has embedded cultural safety in National Law to legislate clinical governance changing racist policies and practices [83]. Change to National Law ‘means that Aboriginal and Torres Strait Islander health consumers can expect that healthcare provision from a registered health practitioner is culturally safe and free of racism’ [83]. This includes foot care. This study is about how non‐Indigenous people working with and for good First Nations foot health need to change ways of working to remove barriers to access to foot care. Mainstream foot health services need to move past praxis relying on deficit‐facing, quantitative, epidemiological data sets of and about First Nations Peoples and First Nations foot health. Such data sets present a constructed understanding of First Nations Peoples as less than, and in need of paternalistic [102], non‐Indigenous saviours for their foot and lower limb health. Existing research documents that strategies privileging First Nations knowledges in First Nations chronic disease care and support ‘should achieve major improvements’ [103]. This study contributes to existing work and commentary in the field of cultural safety and First Nations foot health research [26, 27, 61, 104, 105, 106]. Work brings ways of being and doing in foot health research that can make spaces safer for First Nations Peoples to work in, as judged by them, and that can produce First Nations led ways of knowing how to improve, change, and inform inclusive and responsive foot health systems. This study amplifies perceptions of First Nations and non‐Indigenous health practitioners working with and for First Nations Peoples supporting foot health regarding challenges accessing and barriers to providing good foot health service delivery. Participants' perspectives align with broader reported First Nations health lived experience, guidelines, plans, strategies, and published works [26, 27, 55, 62, 107]. This study provides qualified, voiced lived experience which the podiatry profession must listen to and receive direction and learning from.

## 7 Strengths and Limitations

Strengths are embedded in privileging First Nations knowledges and ways of working in foot health research and clinical practice. This facilitates authentic work bringing attention to health practitioners' perspectives of challenges in accessing, and barriers to providing, good foot care with and for First Nations Peoples. A further strength of this study is that it will inform needs analysis underpinning action and support for First Nations‐led co‐designed foot health services. Concepts and constructs are intra‐connected within this work and are inter‐connected with previous research and discussion presented by the Communities and authors leading this research [63, 108]. A limitation of work is its confinement to the framework of publication; it is not the intention of this study to oversimplify relationality of complex constructs. This publication therefore urges readership to take time in reading, and to make time for further learning, and unlearning. Take time to let considerations sit with oneself, relating to one's positioning to this work.

## 8 Conclusion

First Nations‐led co‐design in ways of working in Aboriginal Participatory Action Research investigated First Nations and non‐Indigenous foot health practitioners' perspectives of challenges in attending and barriers to providing good foot care with and for First Nations Peoples. This study found challenges to attending foot care services for First Nations Peoples were relationships which were not culturally centred, nor longer term, and therefore limited trust. In addition, clinical safety, transport, appointment structures, and different aspirations, behaviours, and ways of working at the cross‐cultural interface present challenges to access. The study also found First Nations disempowerment challenges Communities in accessing support with foot health. This research identified service barriers to providing good foot care with and for First Nations Peoples as distance and funding. Compounding these barriers were lack of health professional cultural capability education and its resulting impacts on clinical services dependant on individuals' behaviours and knowledges, and services' governance. Work identified that time allows for bringing everybody along on a journey improving one's foot health. This study provides qualified, voiced lived experience of health practitioners working in the space to support ongoing efforts to do better. Further work needs to centre the cultures and leadership of First Nations individuals, families, and Communities in working with and for them to keep feet strong.

## Author Contributions

**James Gerrard:** project administration, data curation, formal analysis, writing – original draft, writing – review and editing. **Shirley Godwin (Badimaya Yamatji):** supervision, validation, data curation, formal analysis, visualization, methodology, writing – review and editing. **Kim Whiteley (Wiradjuri):** supervision, validation, formal analysis, visualization, methodology. **James Charles (Kaurna):** supervision, validation, formal analysis, visualization, methodology. **Vivienne Chuter:** supervision, data curation, formal analysis, visualization, validation, methodology, writing – review and editing.

## Funding

The authors have nothing to report.

## Ethics Statement

Ethical approval was granted by The Aboriginal Health and Medical Research Council (1376/18) and the University of Newcastle's Human Research Ethics Committee (approval No. H‐2018 0035).

## Consent

All participants provided written informed consent. The culturally responsive Participant Information Statement received by all participants included information that de‐identified findings would be used in a publication.

## Conflicts of Interest

The authors declare no conflicts of interest.

## Supporting information


Supporting Information S1

Supporting Information S2

## Data Availability

Aligning with Maiam nayri Wingara principles of Indigenous Data Sovereignty and Governance, please contact author for data and related materials requests. The Aboriginal and Torres Strait Islander Quality Appraisal Tool is included as a Supporting Information S2.
