# Supplementary material for: Working Right Ways: Investigating Health Practitioners' Perspectives of Challenges and Barriers to Providing Good Foot Care With and for First Nations Peoples
Source: J Foot Ankle Res. 2026 Jul 23;19(3):e70179. doi: 10.1002/jfa2.70179 (PMC13392920; doi:10.1002/jfa2.70179)
Supplement: Supplementary file 1 — Supporting Information S1 [file JFA2-19-e70179-s002.pdf]

ABORIGINAL AND TORRES STRAIT ISLANDER QUALITY APPRAISAL TOOL

Answer either "Yes", "Partially", "No" or "Unclear" to each question

Article citation: Health practitioners' perspectives of challenges and barriers to providing good foot care with and for First Nations Peoples Date: 26 May 2026

Reviewer's name: Dr Shirley Godwin (Badimaya Yamatji) and James Gerrard

Question	Yes	Partially	No	Unclear
1. Did the research respond to a need or priority determined by the community?	☑	☐	☐	☐
2. Was community consultation and engagement appropriately inclusive?	☑	☐	☐	☐
3. Did the research have Aboriginal and Torres Strait Islander research leadership?	☑	☐	☐	☐
4. Did the research have Aboriginal and Torres Strait Islander governance?	☑	☐	☐	☐
5. Were local community protocols respected and followed?	☑	☐	☐	☐
6. Did the researchers negotiate agreements in regards to rights of access to Aboriginal and Torres Strait Islander peoples' <u>existing</u> intellectual and cultural property?	☑	☐	☐	☐
7. Did the researchers negotiate agreements to protect Aboriginal and Torres Strait Islander peoples' ownership of intellectual and cultural property <u>created</u> through the research?	☑	☐	☐	☐
8. Did Aboriginal and Torres Strait Islander peoples and communities have control over the collection and management of research materials?	☑	☐	☐	☐
9. Was the research guided by an Indigenous research paradigm?	☑	☐	☐	☐
10. Does the research take a strengths-based approach, acknowledging and moving beyond practices that have harmed Aboriginal and Torres Strait peoples in the past?	☑	☐	☐	☐
11. Did the researchers plan and translate the findings into sustainable changes in policy and/or practice?	☑	☐	☐	☐
12. Did the research benefit the participants and Aboriginal and Torres Strait Islander communities?	☑	☐	☐	☐
13. Did the research demonstrate capacity strengthening for Aboriginal and Torres Strait Islander individuals?<br>Whilst no First Nations Peoples or organisations were directly employed or trained as a part of work, First Nations Peoples gained experience in leading and conducting research which does strengthen professional capacity.	☐	☑	☐	☐
14. Did everyone involved in the research have opportunities to learn from each other?	☑	☐	☐	☐
