# Supplementary material for: Working Right Ways: Investigating Health Practitioners' Perspectives of Challenges and Barriers to Providing Good Foot Care With and for First Nations Peoples
Source: J Foot Ankle Res. 2026 Jul 23;19(3):e70179. doi: 10.1002/jfa2.70179 (PMC13392920; doi:10.1002/jfa2.70179)
Supplement: Supplementary file 2 — Supporting Information S2 [file JFA2-19-e70179-s001.pdf]

NHMRC CREATE

The Centre of Research Excellence in Aboriginal Chronic Disease Knowledge Translation and Exchange

The Aboriginal and Torres Strait Islander Quality Appraisal Tool

Companion Document

ACKNOWLEDGEMENT OF COUNTRY

We acknowledge and celebrate that Aboriginal and Torres Strait Islander people are the Traditional Custodians of the land, known as Australia. We recognise that Aboriginal and Torres Strait Islander people are the First Peoples of Australia and that within these two distinct cultural groups, there is great cultural diversity.

We acknowledge that SAHMRI is located on the traditional lands of the Kaurna people and pay our respects to the Kaurna people, Elders, past and present, their continuing connection to this land and thriving cultural practices and knowledge.

© The South Australian Health and Medical Research Institute Limited and The University of Adelaide 2018. All rights reserved.

ISBN-13: 978-0-6480943-9-5

First Published 2018

THE ABORIGINAL AND TORRES STRAIT ISLANDER QUALITY APPRAISAL TOOL: Companion Document

This work is published and disseminated as part of the activities of the Centre of Research Excellence in Aboriginal Chronic Disease Knowledge Translation and Exchange, a National Health and Medical Research Council (NHMRC No 1061242) funded research program.

All rights reserved. No part of this publication may be produced, stored in a retrieval system, or transmitted in any form or by any means, electronic, mechanical, photocopying, recording or otherwise, without the prior permission of the Centre of Research Excellence in Aboriginal Chronic Disease Knowledge Translation and Exchange.

Requests and enquiries concerning reproduction and rights should be addressed to the email address below.

The Centre of Research Excellence in Aboriginal Chronic Disease Knowledge Translation and Exchange
South Australian Health and Medical Research Institute, Adelaide, South Australia 5000
Email: infocreate@sahmri.com
Web: https://create.sahmri.org/

Suggested citation: Harfield S, Pearson O, Morey K, Kite E, Glover K, Canuto K, Streak Gomersall J, Carter D, Davy C, Aromataris E, Braunack-Mayer A. The Aboriginal and Torres Strait Islander Quality Appraisal Tool: Companion Document. Adelaide, Australia: South Australian Health and Medical Research Institute, 2018.

CONTENTS

Centre of Research Excellence in Aboriginal Chronic Disease Knowledge Translation and Exchange **4**

Preamble **5**

What is the Aboriginal and Torres Strait Islander Quality Appraisal Tool? 5
What is the Aboriginal and Torres Strait Islander Quality Appraisal Tool Companion Document? 5
What is the purpose of the Quality Appraisal tool? 5
Why do we need the Aboriginal and Torres Strait Islander Quality Appraisal Tool? 5
How was the Aboriginal and Torres Strait Islander Quality Appraisal Tool Developed? 6
How to use the Aboriginal and Torres Strait Islander Quality Appraisal Tool? 6

Definitions **7**

Aboriginal and Torres Strait Islander Quality Appraisal Tool **8**

Did the research respond to a need or priority determined by the community? 9
Was community consultation and engagement appropriately inclusive? 10
Did the research have Aboriginal and Torres Strait Islander research leadership? 11
Did the research have Aboriginal and Torres Strait Islander governance? 12
Were local community protocols respected and followed? 13
Did the researchers negotiate agreements in regards to rights of access to existing Aboriginal and Torres Strait Islander peoples' intellectual and cultural property? 14
Did the researchers negotiate agreements to protect the intellectual and cultural property of Aboriginal and Torres Strait Islander peoples created through the research? 15
Did Aboriginal and Torres Strait Islander peoples and communities have control over the collection and management of research materials? 16
Was the research guided by an Indigenous research paradigm? 17
Does the research take a strengths based approach, acknowledging and moving beyond practices that have harmed Aboriginal and Torres Strait peoples in the past? 18
Did the researchers plan and translate the findings into sustainable changes in policy and/or practice? 19
Did the research benefit the participants and Aboriginal and Torres Strait Islander communities? 20
Did the research demonstrate capacity strengthening for Aboriginal and Torres Strait Islander individuals? 21
Did everyone involved in the research have opportunities to learn from each other? 22

References **23**

CENTRE OF RESEARCH EXCELLENCE IN ABORIGINAL CHRONIC DISEASE KNOWLEDGE TRANSLATION AND EXCHANGE

The Centre of Research Excellence in Aboriginal Chronic Disease Knowledge Translation and Exchange (CREATE) - is a National Health and Medical Research Council (NHMRC) funded research program dedicated to improving service delivery and health outcomes for Aboriginal and Torres Strait Islander peoples, with a particular focus on chronic disease. The Centre is a collaborative enterprise between:

- National Aboriginal Community Controlled Health Organisation (NACCHO)
- Wardliparingga Aboriginal Research Unit, South Australian Health and Medical Research Institute (SAHMRI);
- University of Adelaide – School of Public Health and Joanna Briggs Institute

CREATE aims to assist the Aboriginal community controlled health sector to improve the coverage and appropriateness of its services and care through the synthesis of new and existing knowledge (published and unpublished literature) about best-practice chronic disease prevention and management as well as through the creation of sustainable primary health care funding and service delivery models.

Objectives:

- To use existing evidence and, where necessary, develop and collate new evidence to inform guidelines, policies and/or other tools focused on improving care and outcomes experienced by Aboriginal and Torres Strait Islander peoples with, or at risk of developing, a chronic disease.
- To strengthen the capacity of Aboriginal and Torres Strait Islander health service providers and researchers to conduct and use evidence to improve health outcomes.

PREAMBLE

What is the Aboriginal and Torres Strait Islander Quality Appraisal Tool?

The ***Aboriginal and Torres Strait Islander Quality Appraisal Tool*** is a set of fourteen questions for appraising the quality of research in Australian settings with Aboriginal and Torres Strait Islander peoples, families and communities through an Aboriginal and Torres Strait Islander lens. Aboriginal and Torres Strait Islander peoples' values and ethics have informed the questions in the tool with the intent to achieve appropriate, high quality and relevant health research that benefits Aboriginal and Torres Strait Islander peoples.

What is the Aboriginal and Torres Strait Islander Quality Appraisal Tool Companion Document?

The ***Aboriginal and Torres Strait Islander Quality Appraisal Tool Companion Document*** provides users with guidance on how to interpret and assess research articles using the ***Aboriginal and Torres Strait Islander Quality Appraisal Tool.***

What is the purpose of the Quality Appraisal Tool?

The ***Aboriginal and Torres Strait Islander Quality Appraisal Tool*** is designed to appraise the quality of studies, primarily as part of the systematic review process. It has been designed to consider studies in Australian settings with Aboriginal and Torres Strait Islander peoples, families and communities. Other users of the ***Aboriginal and Torres Strait Islander Quality Appraisal Tool*** may include: 1) editors of journals that include studies involving Aboriginal and Torres Strait Islander participants; 2) reviewers of journal articles reporting Aboriginal and Torres Strait Islander research; 3) funders who review proposals for research studies involving Aboriginal and Torres Strait Islander participants; and 4) researchers planning to carry out research with Aboriginal and Torres Strait Islander peoples. The tool is designed to be used in addition to other critical appraisal tools.

Why do we need the Aboriginal and Torres Strait Islander Quality Appraisal Tool?

The history of colonisation of Aboriginal and Torres Strait Islander peoples in Australia is reflected in the record of research with Australia's culturally diverse Aboriginal and Torres Strait Islander peoples.[1-3] Health and medical research in particular, has a long record of researchers gathering information from Aboriginal and Torres Strait Islander peoples without consulting and gaining approval from relevant Aboriginal and Torres Strait Islander peoples and organisations.[2] Rather than working in partnership with Aboriginal and Torres Strait Islander research participants and being guided by Aboriginal and Torres Strait Islander peoples, researchers have tended to treat participants as research objects.[2] Western research values and Western ontology, epistemology and axiology have governed the methodologies that have dominated health research in Australia.[2, 4] Informed by Western research values, researchers have defined the objectives of research without foremost considering the research participants and their communities' needs. Western research methodologies are fundamentally different from those of Aboriginal and Torres Strait Islander peoples, whose ways of knowing, being and doing are based on

lived experiences and knowledge as Aboriginal and Torres Strait Islander peoples. Reliance on Western ways of doing health research in Australian settings with Aboriginal and Torres Strait Islander participants has produced research that has exploited and harmed Aboriginal and Torres Strait Islander peoples and prevented research from being an effective tool to improve the health and wellbeing of Aboriginal and Torres Strait Islander peoples. Recent research has begun to take a strengths-based approach, ensuring research is conducted with and for the benefit of Aboriginal and Torres Strait Islander peoples.

How was the Aboriginal and Torres Strait Islander Quality Appraisal Tool Developed?

Senior Aboriginal and Torres Strait Islander health researchers, together with ethicists and systematic review experts, developed the tool and companion document over a three-year period, using a combination of literature review and interactive group work. A modified Delphi method was used to assess the face validity, reliability and feasibility of the tool. An independent Australian panel comprising Aboriginal and Torres Strait Islander researchers critiqued the tool and made recommendations for improvements. Systematic reviewers independent of the development then trialed the tool for reliability and feasibility.

How to use the Aboriginal and Torres Strait Islander Quality Appraisal Tool?

The ***Aboriginal and Torres Strait Islander Quality Appraisal Tool*** should be applied to articles that report research involving or related to Aboriginal and Torres Strait Islander peoples, families and communities. The ***Aboriginal and Torres Strait Islander Quality Appraisal Tool*** should be used in conjunction with existing tools to critically appraise research. This will enable a more comprehensive assessment of study quality and value by including review through an Aboriginal and Torres Strait Islander lens.

Each of the 14 questions in the ***Aboriginal and Torres Strait Islander Quality Appraisal Tool*** should be used to assess evidence contained within the article. The examples included in this Companion Document illustrate good practice in relation to each question and should be used as a guide. In addition, separate correspondence that has been specifically sought for clarification from the author should also be considered in assessing quality.

If the article provides adequate evidence to answer ‘yes’ to a question, then “Yes” should be marked. If the answer to a question is ‘partially’, then, “Partially” should be marked. If there is no evidence that the answer to a question is ‘no’, then “No” should marked. If the answer to a question is unclear, including when there is no evidence in the article to answer a question, then “Unclear” should be marked.

The appraisal of each paper may be summarised by the number of “Yes”, “No”, “Partial” and “Unclear” answers or displayed in a table. Further discussion on the appraisal may also be provided.

N.B. – Answering ‘yes’ to all of the questions in the ***Aboriginal and Torres Strait Islander Quality Appraisal Tool*** does not negate the need to ensure appropriate ethics approval has been received prior to the commencement of any study. Research with Aboriginal and Torres Strait Islander peoples, communities and organisations must receive ethics approval from an Aboriginal Human Research Ethics Committee. This ensures that the research aligns with ethical guidelines such as the National Health and Medical Research Council Road Map 3: A strategic framework for improving Aboriginal and Torres Strait Islander health through research [5], Values and Ethics: Guidelines for Ethical Conduct in Aboriginal and Torres Strait Islander Health Research[6] and the National Statement on Ethical Conduct in Human Research[7]. It also demonstrates that appropriate consideration has been given to the conception, design and conduct of research with Aboriginal and Torres Strait Islander communities.

DEFINITIONS

While there are more conventional definitions for the following words and phrases, the definitions provided below are specific to the context of conducting research with Aboriginal and Torres Strait Islander peoples, families and communities.

BENEFIT

The benefits of the research must be determined by the Aboriginal and Torres Strait Islander community participating in the research. The type of benefit will vary depending on the research and the participants involved in the research, but it should be meaningful, appropriate and proportional to participant involvement.

CAPACITY STRENGTHENING

Aboriginal and Torres Strait Islander peoples and communities are to be provided training and employment opportunities throughout the research project. At the end of the project, Aboriginal and Torres Strait Islander peoples and communities should have additional skills, experience and knowledge to negotiate, assist with, implement and lead future research.

COMMUNITY

A group of people living in the same place or belonging to the same language group or having a particular characteristic in common, at an organisational, local, state or national level.

CONTROL

The power to influence or direct people's behaviour or the course of research processes and outcomes.

CULTURAL AND INTELLECTUAL PROPERTY

The rights of Aboriginal and Torres Strait Islander peoples and communities to share, access, control, maintain and grow their cultural and intellectual heritage. Cultural and intellectual property includes the tangible and intangible, including knowledge, artefacts and expression. It incorporates all aspects of knowledge (sciences, plant and animal knowledge, stories, designs and symbols, ritual knowledge), artefacts (arts, crafts, weapons, tools and technology), expression (ceremonies, dance and song) and human remains, and includes the secret and sacred. These rights are perpetual and form a living heritage, reinterpreted by each new generation [8]. It is used to inform the research or generated from the research.

FAMILY

A group of two or more people who are either immediate or extended family, and who identify as a family.

GOVERNANCE

An existing or established group or organisation that enables and monitors the implementation of community protocols, provides the relevant cultural and contextual knowledge to inform the research, and enables Aboriginal and Torres Strait Islander peoples to have authority over the research throughout the research process.

INDIGENOUS RESEARCH PARADIGM

An approach that reflects Aboriginal and/or Torres Strait Islander ways of knowing, being and doing [4, 8, 9] and is based on the lived experiences and knowledges of Aboriginal and Torres Strait Islander peoples. It informs and guides the research processes.

ABORIGINAL AND TORRES STRAIT ISLANDER QUALITY APPRAISAL TOOL

CREATE

ABORIGINAL AND TORRES STRAIT ISLANDER QUALITY APPRAISAL TOOL

Answer either “Yes”, “Partially”, “No” or “Unclear” to each question

Article citation: ____ Date: ______________________

Reviewer’s name: ____

Question	Yes	Partially	No	Unclear
1. Did the research respond to a need or priority determined by the community?				
2. Was community consultation and engagement appropriately inclusive?				
3. Did the research have Aboriginal and Torres Strait Islander research leadership?				
4. Did the research have Aboriginal and Torres Strait Islander governance?				
5. Were local community protocols respected and followed?				
6. Did the researchers negotiate agreements in regards to rights of access to Aboriginal and Torres Strait Islander peoples’ existing intellectual and cultural property?				
7. Did the researchers negotiate agreements to protect Aboriginal and Torres Strait Islander peoples' ownership of intellectual and cultural property created through the research?				
8. Did Aboriginal and Torres Strait Islander peoples and communities have control over the collection and management of research materials?				
9. Was the research guided by an Indigenous research paradigm?				
10. Does the research take a strengths-based approach, acknowledging and moving beyond practices that have harmed Aboriginal and Torres Strait peoples in the past?				
11. Did the researchers plan and translate the findings into sustainable changes in policy and/or practice?				
12. Did the research benefit the participants and Aboriginal and Torres Strait Islander communities?				
13. Did the research demonstrate capacity strengthening for Aboriginal and Torres Strait Islander individuals?				
14. Did everyone involved in the research have opportunities to learn from each other?				

Did the research respond to a need or priority determined by the community?

The research effort should respond to priorities arising from, negotiated with and endorsed by the Aboriginal and Torres Strait Islander community affected by the research. This will ensure the research is relevant and improve its acceptability and accountability to the Aboriginal and Torres Strait Islander community that will be impacted.

Things to look for in written documentation:

- An Aboriginal or Torres Strait Islander community, group or organisation approached the researcher or research group with a research question or project.
- An Aboriginal or Torres Strait Islander community, group or organisation was involved in identifying and setting priorities.
- Priorities were identified through national, state or local documents, such as policies, plans and strategies e.g. National Indigenous Reform Agreement, The National Aboriginal and Torres Strait Islander Health Plan.
- Emerging issues that impact on Aboriginal and Torres Strait Islander peoples, families and communities both politically and socially are recognised by the communities themselves.

PRACTICAL EXAMPLES

An Aboriginal health organisation approached researchers with an idea for a research project on sexual health screening within their community.
A community forum was held to identify and set priorities about an emerging issue faced by an Aboriginal community.
At a national conference on Aboriginal and Torres Strait Islander mental health, participants called upon governments to address the prevalence of suicide among Aboriginal and Torres Strait Islander peoples.
An Aboriginal community, group or organisation has been advocating for cheaper and healthier food and drink items at their local community store.

Was community consultation and engagement appropriately inclusive?

Significant diversity exists within and across Aboriginal and Torres Strait Islander communities. Therefore generalisations cannot be made. Engagement and inclusion of a range of existing organisations, groups and governance structures within the local community prior to and throughout the research is appropriate and good practice.

Things to look for in the written documentation:

- Evidence that organisations representing the participants were consulted prior to starting the research.
- Evidence that researchers identified and consulted a diverse range of relevant local community organisations and groups to cover the range of interests and needs of the research participants.
- Statements by authors about a change in the question, method, interpretation of results or knowledge translation based on consultation with the community.

PRACTICAL EXAMPLES

Prior to starting a large cohort study, consultation occurred with every Aboriginal community throughout the state. A second round of consultation occurred before the start of each community site and the recruitment of participants. It was during the initial consultations that it became apparent that the original name of the study had no meaning to Aboriginal peoples and therefore the name was changed to reflect what the study was investigating.

Did the research have Aboriginal and Torres Strait Islander research leadership?

It is expected that research with Aboriginal and Torres Strait Islander peoples, families and communities has Aboriginal and Torres Strait Islander leadership. Ideally, an Aboriginal and Torres Strait Islander person would be a principal investigator or, at the very least, a member of the research leadership team. Having Aboriginal and Torres Strait Islander persons as principal or senior investigators helps to ensure that Aboriginal and Torres Strait Islander peoples' ways of knowing, being and doing are reflected throughout the research project, and that the research aligns with the National Health and Medical Research Council Road Map 3: A strategic framework for improving Aboriginal and Torres Strait Islander health through research [5] and *Values and Ethics: Guidelines for Ethical Conduct in Aboriginal and Torres Strait Islander Health Research*.[6]

Things to look for in the written documentation:

- One or more of the Chief Investigators is an Aboriginal and/or Torres Strait Islander person.
- An Aboriginal and/or Torres Strait Islander person or group of people led the research implementation process.
- The research team consists of Aboriginal and/or Torres Strait Islander people who are responsible to the Aboriginal or Torres Strait Islander community for the integrity of the research and its output. These people have the authority to make decisions and may be referred to as senior researchers or senior Aboriginal leaders.

PRACTICAL EXAMPLES

One or more Aboriginal researchers are Chief Investigators of a Centre of Research Excellence.
A group of Aboriginal and Torres Strait Islander researchers led the development of an appraisal tool which is used to assess the quality of research involving Aboriginal and Torres Strait Islander peoples.
A research project investigating the benefits of dietary supplements within Aboriginal and Torres Strait Islander populations is administered by an Aboriginal Research Nurse.

Did the research have Aboriginal and Torres Strait Islander governance?

There should be Aboriginal and Torres Strait Islander authority over the research throughout the research process. Aboriginal and/or Torres Strait Islander governance structures ensure community protocols are followed and enable relevant cultural and contextual knowledge to inform the research. Researchers must work together with relevant Aboriginal and/or Torres Strait Islander peoples throughout the research process. Researchers must work with a relevant existing governance structure or establish one or more Aboriginal and Torres Strait Islander governance structure.

Things to look for in written documentation:

- Evidence that a group of Aboriginal and/or Torres Strait Islander peoples informed, guided, monitored and had some degree of control over the research throughout the research process, including in planning stages.
- Evidence that the researchers reported to the group of Aboriginal and/or Torres Strait Islander peoples, that their guidance informed the direction of the project, and that the governance structure had authority to make decisions.

PRACTICAL EXAMPLES

A community reference group was established with members representing each of the communities involved in the research project. The community reference group guided and had authority over the research throughout the research process to ensure the research was conducted appropriately, and with meaningful analysis, interpretation and outcomes.

Were local community protocols respected and followed?

Community obligations and protocols will always take precedence over formal business such as research. In the case of unexpected events, community leaders and members will be required to meet their cultural obligations, which may impact upon the research timelines and outcomes. Protocols may differ between local communities and it is the responsibility of researchers to familiarise themselves with and to follow the appropriate local protocols of the community before engagement commences. This will reduce the risk of the researcher breaching local community protocols.

Things to look for in written documentation:

- Evidence that local protocols were followed to access appropriate research participants, for example females for women's business research.
- Evidence that timing of the research was changed to respect significant community events, for example the need for community members to participate in sorry business.
- Evidence that the researchers aligned their data collection approach to ensure that cultural protocols of the community were respected.
- Evidence that interpreters were used to ensure community protocol was adhered to where English is not the first language.

PRACTICAL EXAMPLES

Cultural knowledge was shared with the researcher to inform the research; however, specific cultural knowledge content were not included in any research outcomes as it was not appropriate to or approved by the leaders of the community.
Due to a significant unplanned event in the community there was a delay in the research.

Did the researchers negotiate agreements in regards to rights of access to Aboriginal and Torres Strait Islander peoples' existing intellectual and cultural property?

In all research projects a formal agreement should be negotiated, outlining the rights of access to Aboriginal and Torres Strait Islander peoples' existing intellectual and cultural property, including acknowledging the contribution of Aboriginal and Torres Strait Islander peoples. Whilst Memoranda of Understanding (MOUs) are commonly used to document agreements about the rights and responsibilities of the partners in research, a legally binding agreement is preferred, as this is better able to protect and promote the interests of Aboriginal and Torres Strait Islander peoples. The agreement should clarify the roles and responsibilities of the researchers and community members in the research and it should describe the benefits to the community as determined by community, including in terms of resource sharing and training to be delivered as part of the research. It should also describe the rights of access to Aboriginal and Torres Strait Islander peoples' intellectual and cultural property and data ownership.

Things to look for in written documentation:

- A statement in the article that a legally binding contract to protect the intellectual and cultural property of the participants and community involved was developed and negotiated between the researcher and the responsible community organisations and individuals prior to the research.

PRACTICAL EXAMPLES

A Collaborative Research Agreement was developed in partnership between the research institute and the local community board. The Agreement outlined the roles and responsibilities of the two organisations with regard to the sharing of resources, consultation and engagement, cultural protocols, research outcomes, research benefits for the community, employment and training opportunities, timeframes and existing intellectual and cultural property rights.
A researcher writing a biography of an Aboriginal artist negotiated an agreement with the artist and their community.

Did the researchers negotiate agreements to protect Aboriginal and Torres Strait Islander peoples' ownership of intellectual and cultural property created through the research?

Knowledge created through research must remain the intellectual property of Aboriginal and Torres Strait Islander contributors, and all published material must abide by the National Health and Medical Research Council's *Values and Ethics: Guidelines for Ethical Conduct in Aboriginal and Torres Strait Islander Health Research*.[6] Researchers must appropriately acknowledge in research outputs the contributions of the Aboriginal and Torres Strait Islander peoples, researchers, research participants and governance bodies in the generation of new knowledge.

Things to look for in written documentation:

- Evidence that the researchers understand that the knowledge generated by the research remains the intellectual property of the Aboriginal and Torres Strait Islander contributors to the research.
- A statement in the article that the knowledge generated by the research is the intellectual property of Aboriginal and Torres Strait Islander peoples involved in the research.

PRACTICAL EXAMPLES

A Collaborative Research Agreement was developed detailing arrangements for the knowledge and intellectual property generated from the research and for the ongoing management and use of that knowledge and intellectual property.
A series of case studies with Aboriginal Community Controlled Health Organisations are prepared. The findings from the research belong to the ACCHO and approval is sought from the ACCHO to publish the findings.

Did Aboriginal and Torres Strait Islander peoples and communities have control over the collection and management of research materials?

Aboriginal and Torres Strait Islander peoples, families and communities must have control over the respectful and appropriate collection and management of all biological and non-biological research materials. Local Aboriginal and Torres Strait Islander communities remain the owners of the data they provide as research participants.

Things to look for in written documentation:

- Development of a protocol in partnership with Aboriginal and Torres Strait Islander peoples for the respectful and appropriate collection and management of all biological and non-biological research materials.
- The consent processes give the participants control over how their data and samples will be managed.
- Evidence that participant consent is specific to the project and not for extended or unspecified uses.

PRACTICAL EXAMPLES

Protocols relating to the collection, use, management and storage of data were developed in partnership with Aboriginal and Torres Strait Islander people and adhered to by researchers.
A community withheld information relating to private cultural knowledge and practices because it thought that the information might harm the community due to its political sensitivity. This was reported as an aspect of the research process that was cancelled due to advice from community leaders.
Aboriginal and Torres Strait Islander research leadership contributed to Aboriginal and Torres Strait Islander control of data.
The public findings from the study included a statement about not publishing, withholding, withdrawing or destroying data (this may be in the form of biological samples or health information).

Was the research guided by an Indigenous research paradigm?

An Aboriginal or Torres Strait Islander research paradigm reflects Aboriginal and/or Torres Strait Islander ways of knowing, being and doing [4, 9, 10] and is based on the lived experiences and knowledges of Aboriginal and Torres Strait Islander peoples. The research methodology and methods must reflect the community values, priorities and perspectives of research participants and their communities.

Diversity exists within and across Aboriginal and Torres Strait Islander communities in Australia. It is the responsibility of the researchers to ensure that the diversity is understood, protected, respected and reflected within the research process. The use of unique and local knowledge is critical to ensure that the data collection, analysis and interpretation include all of the issues that are important for making valid conclusions and relevant recommendations.

Things to look for in written documentation:

- Evidence that an Indigenous research paradigm was used, with a clear description of how it reflects Aboriginal and Torres Strait Islander ways of knowing, being and doing.
- Acknowledgment that health and wellbeing are complex and interconnected and require multiple research methods.
- Evidence that the ways of knowing, being and doing of the Aboriginal and Torres Strait Islander peoples informed research processes, including engagement, conceptualisation, implementation, interpretation and dissemination of research findings.
- A description of steps taken by the researchers, prior to and during the research, to understand the perspectives of relevant local cultural and contextual experts (for example, Elders, board members, local community groups), together with a description of how this knowledge was integral to the research.

PRACTICAL EXAMPLES

'This study used an exploratory, descriptive design, guided by an Indigenous research approach. The research question being asked, the participants being interviewed, as well as one of the Chief Investigators identifying as being Aboriginal and/or Torres Strait Islander, demanded the use of a research approach that removes the voice of the coloniser and places value on Indigenous knowledge. The research team believed it was important to privilege Indigenous voices and Indigenous lives.'[11]
An Aboriginal female researcher using a mixed-method design to assess prevalence and experiences of asthma amongst Aboriginal youth identified herself as a member of the Aboriginal community and described how being part of this community shaped her research methodology.
The use of state and national level data sets to report and monitor health inequalities at a local level, with the local community proposing research questions, informing analysis and interpreting results.

Does the research take a strengths-based approach, acknowledging and moving beyond practices that have harmed Aboriginal and Torres Strait peoples in the past?

Researchers and research practices must build on Aboriginal and Torres Strait Islander strengths and resilience. Research must contribute to improved health, social and economic outcomes experienced by Aboriginal and Torres Strait Islander peoples, families and communities. Researchers and research processes must avoid practices which have been harmful to Aboriginal and Torres Strait Islander peoples, families and communities.

Things to look for in written documentation:

- Evidence that the researchers acknowledge past harms, make efforts not to replicate past practices, and understand that the imposition of Western values and perspectives is detrimental to Aboriginal and Torres Strait Islander peoples' health.
- The research takes a strengths-based approach by identifying positive attributes such as resilience as opposed to risk factors that are already established.
- Evidence that the research identified and built upon strengths in the local community of the research participants and/or strengths of other Aboriginal and Torres Strait Islander communities.

PRACTICAL EXAMPLES

The researchers acknowledged that in certain circumstances past research practices have done more harm than good and have impacted negatively on the health and wellbeing of Aboriginal and Torres Strait Islander peoples, families and their communities.
The research question on mental health was altered to take a strengths-based approach and to avoid a deficit approach.
Findings of a research project outlined the protective factors of maintaining connection to Country that contribute to positive health and wellbeing outcomes.
Research offers solutions that have been informed by Aboriginal and Torres Strait Islander community members and the findings of the research.

Did the researchers plan to and translate the findings into sustainable changes in policy and/or practice?

It is essential that researchers disseminate research processes or findings to relevant individuals and organisations so as to contribute to sustainable improvements in policy or practice. This requires planning at the outset. Research on Aboriginal and Torres Strait Islander priorities, done in partnership with the community, is more likely to bring about positive change.

Things to look for in written documentation:

- Evidence of a comprehensive knowledge-translation plan which has been implemented.
- Evidence that the research has resulted in policy development or informed practice.
- Evidence that skills transferred to Aboriginal and Torres Strait Islander peoples through the research are likely to lead to sustainable changes in the way health policy is formulated, the content of health policies, health service delivery, or the health of Aboriginal and Torres Strait Islander peoples.

PRACTICAL EXAMPLES

Research governed by an Aboriginal Reference Group whose role included interpreting both epidemiological and qualitative findings was used to inform the development of a state-wide Aboriginal policy document and patient-reported outcome measure, both of which were used to inform clinical care for Aboriginal peoples.

Did the research benefit the participants and Aboriginal and Torres Strait Islander communities?

Research must produce meaningful benefits for Aboriginal and Torres Strait Islander peoples and their communities. It must do more than merely describe the issues. The benefits of the research must be determined by the Aboriginal and Torres Strait Islander community that is participating in the research.

Things to look for in written documentation:

- Evidence that research provided a service - "No survey without service" - to the Aboriginal and Torres Strait Islander participants.
- Evidence that the research provided a resource for the local Aboriginal and Torres Strait community, where the research was being done.
- Evidence that the research outcomes also benefited Aboriginal and Torres Strait Islander peoples belonging to other communities.
- Authors reported the short-, medium- and long-term benefits (as identified by the community) that the research delivered to Aboriginal and Torres Strait Islander people and/or the local community.

PRACTICAL EXAMPLES

A cohort study involving the delivery of a screening program provided individuals with an immediate clinical service, treated treatable conditions and facilitated ongoing care through clinical integration.
A research project focusing on environmental health resulted in a water fountain being installed in three community parks.
A tool was developed and subsequently used to help guide future research with Aboriginal and Torres Strait Islander peoples in a culturally responsive and safe way.

Did the research demonstrate capacity strengthening for Aboriginal and Torres Strait Islander individuals?

Aboriginal and Torres Strait Islander peoples should be trained and employed throughout the research project. Investing in Aboriginal and Torres Strait Islander peoples who are members of the participating communities is essential to improving their health and wellbeing. At the conclusion of the research project, local Aboriginal and Torres Strait Islander peoples should have additional skills, experience and knowledge about how to negotiate, assist with, implement and lead future research. These strengthened attributes will contribute to the advancement of local communities and the broader Aboriginal and Torres Strait Islander community in Australia.

Things to look for in written documentation:

- Evidence of employment of Aboriginal and Torres Strait Islander peoples who are members of the participating community where the research was being done.
- Evidence that Aboriginal and Torres Strait Islander peoples employed in the research continue with other research roles.
- Evidence of formal or informal training of Aboriginal and Torres Strait Islander peoples delivered as part of the research process.
- Evidence that local businesses owned by or employing Aboriginal and Torres Strait Islander peoples were used to provide services for research activities.

PRACTICAL EXAMPLES

Local Aboriginal and Torres Strait Islander Health Workers were seconded part time to work as research officers on an 18-month project whilst being supported through a certified introduction-to-research course.
A local Aboriginal artist was commissioned to develop the research project logo.
A local business owned and operated by Aboriginal people was hired to cater for a research event.
The research itself focused on capacity strengthening; for example, Aboriginal and Torres Strait Islander people were supported through research scholarships to complete an Honours, Masters by research or PhD qualification.
An existing Aboriginal women's group partnered with a research team on a family resilience project. Through the project the Aboriginal women's group linked with other state and national community groups and built on their research track record.

Did everyone involved in the research have opportunities to learn from each other?

There should be two-way learning through the research process that encompasses capacity strengthening for Aboriginal and Torres Strait Islander peoples, families and communities and non-Indigenous and Aboriginal and Torres Strait Islander researchers. Aboriginal and Torres Strait Islander peoples, families and communities should have the opportunity to learn about all components of the research process. Non-Indigenous researchers and their research communities should be able to learn from research with Aboriginal and Torres Strait Islander researchers and participants about their culture and ways of knowing, being and doing.

Things to look for in written documentation:

- Evidence of meaningful partnership between Aboriginal and Torres Strait Islander researchers and non-Indigenous researchers.
- The employment of Aboriginal and Torres Strait Islander peoples on the research project - in research training roles or as researchers, research assistants or culture brokers.
- Researchers spent time at the beginning of the research process with the community to understand community protocols and the culture of participants and shared their knowledge about the research process.
- Researchers presented the findings back to the participants and the community at the end of the research project.

PRACTICAL EXAMPLES

A cross-sectional population-based study being led by a non-Indigenous principal researcher with the aim of investigating Aboriginal women's health undertook extensive pre-planning consultation, which resulted in the establishment of an Aboriginal Advisory Group to guide the conduct of consultations and development of the research. The Aboriginal Advisory Group members were active partners in the research from its inception and provided leadership, guidance and direction to the project and were all investigators of the study in their own right. Through this, the principal researcher grew in their capacity to conduct research with Aboriginal communities the right way.

REFERENCES

1. Australian Institute of Aboriginal and Torres Strait Islander Studies and The Lowitja Institute, *Researching Right Way: Aboriginal and Torres Strait Islander Health Research Ethics: A Domestic and International Review*. 2013, Cooperative Research Centre for Aboriginal and Torres Strait Islander Health: Melbourne.

2. Laycock, A., et al., *Researching Indigenous Health: A practical guide for researchers*. 2011, The Lowitja Institute: Melbourne.

3. Rigney, L.-I., *Internationalization of an Indigenous Anticolonial Cultural Critique of Research Methodologies: A Guide to Indigenist Research Methodology and Its Principles.* Wicazo Sa Review, 1999. **14**(2): p. 109-121.

4. Kite, E. and C. Davy, *Using Indigenist and Indigenous methodologies to connect to deeper understandings of Aboriginal and Torres Strait Islander peoples' quality of life.* Health Promotion Journal of Australia, 2015. **26**(3): p. 191-194.

5. National Health and Medical Research Council, *Road Map 3: A strategic framework for improving Aboriginal and Torres Strait Islander health through research*. 2018, Commonwealth of Australia: Canberra.

6. National Health and Medical Research Council, *Values and Ethics: Guidelines for Ethical Conduct in Aboriginal and Torres Strait Islander Health Research*. 2003, Commonwealth of Australia: Canberra.

7. National Health and Medical Research Council, *National Statement on Ethical Conduct in Human Research*. 2007 (Updated May 2015), Commonwealth of Australia: Canberra.

8. National Museum of Australia. *Indigenous cultural rights and engagement principles*. 2015 17/08/2017]; Available from: http://www.nma.gov.au/about_us/ips/procedures/indigenous_cultural_rights_and_engagement_principles.

9. Kovach, M.E., *Indigenous methodologies: characteristics, conversations, and contexts* 2010, Toronto: University of Toronto Press.

10. Wilson, S., *Research is ceremony: Indigenous research methods*. 2008, Winnipeg: Fernwood.

11. Kelly, J., et al., *'She knows how we feel': Australian Aboriginal and Torres Strait Islander childbearing women's experience of Continuity of Care with an Australian Aboriginal and Torres Strait Islander midwifery student.* Women and Birth, 2014. **27**(3): p. 157-162.

SAHMRI
South Australian Health & Medical Research Institute

SAHMRI North Terrace, Adelaide, SA, 5000, Australia
P +61 (0)8 8128 4000 **E** reception@sahmri.com
PO Box 11060, Adelaide SA 5001
www.facebook.com/sahmri www.twitter.com/sahmriAU
www.sahmri.org
